# Effect of different post-curing times on the physicomechanical properties of 3D-printed resins intended for permanent restorations

**DOI:** 10.4317/jced.64561

**Published:** 2026-09-15

**Authors:** José Giancarlo Tozo-Burgos, Marco Sánchez-Tito, John Alexis-Domínguez

**Affiliations:** 1 Faculty of Stomatology, Universidad Peruana Cayetano Heredia; 2 2 Research Group on Dental Biomaterials and Natural Products, Faculty of Health Sciences, Universidad Privada de Tacna, Tacna, Perú; 3 Research Group on Dental Biomaterials and Natural Products, Faculty of Health Sciences, Universidad Privada de Tacna, Tacna, Perú

**Keywords:** Additive manufacturing, mechanical properties, 3D printing, post-curing, materials testing.

## Abstract

**Background:**

Three-dimensional (3D)-printed resins intended for permanent restorations represent a promising alternative to conventional restorative materials. Although the effect of post-curing duration on these materials has already been investigated, material-specific evidence for some recently developed resins remains limited. This study aimed to evaluate the effect of different post-curing times on the surface microhardness, flexural strength, and fracture resistance of these resins.

**Materials and Methods:**

An in vitro experimental study was conducted using three 3D-printed resins (VarseoSmile TriniQ, Prizma 3D Bio Crown Diamond, and Prizma 3D Bio Crown) and a CAD/CAM nanohybrid resin (Evolux Hybrid) as the reference group. The printed resins were subjected to three post-curing times: Half the manufacturer-recommended time, the recommended time, and twice the recommended time. Surface microhardness, flexural strength, and fracture resistance were evaluated. Data were analyzed using two-way analysis of variance and the Bonferroni post hoc test (α=0.05).

**Results:**

Material type, post-curing time, and their interaction significantly affected the physicomechanical properties evaluated (p<0.05). For VMH, the highest values were recorded for Bio Crown Diamond-T3 and VarseoSmile TriniQ-T3, at 23.59 ± 1.58 and 23.47 ± 1.17, respectively, with no significant difference between them. VarseoSmile TriniQ-T3 exhibited the highest flexural strength and fracture resistance values, reaching 128.14 ± 11.35 MPa and 933.14 ± 20.77 N, respectively. Bio Crown showed the lowest values for all three properties evaluated.

**Conclusions:**

Post-curing time influenced the physicomechanical properties of 3D-printed resins intended for permanent restorations, with the response depending on the material and property evaluated. VarseoSmile TriniQ exhibited the highest flexural strength and fracture resistance. In contrast, Bio Crown Diamond achieved surface microhardness comparable to that of VarseoSmile TriniQ only after post-curing for twice the recommended duration.

## Introduction

Digital dentistry is now integral to clinical practice due to its ability to enhance restoration accuracy, optimize clinical workflows, and increase the efficiency and predictability of restorative procedures. These benefits are realized through both subtractive manufacturing technologies, such as computer-aided design and computer-aided manufacturing (CAD/CAM), and additive manufacturing technologies, such as three-dimensional (3D) printing [1]. Additive manufacturing via 3D printing has attracted significant attention for its ability to efficiently fabricate complex, customized structures while minimizing material waste compared to subtractive methods. Ongoing advancements in materials, software, and printing technologies have broadened the application of these methods across multiple dental specialties [2].

The primary 3D-printing technologies utilized in dentistry are stereolithography (SLA), digital light processing (DLP), selective laser sintering (SLS), and fused deposition modeling (FDM) [3,4]. Of these, SLA and DLP systems are most commonly employed in dental practice because of their superior resolution [5]. Initial dental applications of 3D printing focused on fabricating working models, surgical guides, and provisional restorations [6]. Recent advancements in resin formulations, particularly the incorporation of inorganic fillers such as silica and zirconia particles, have enabled the development of materials with enhanced physicomechanical properties [7].

In this context, Bora *et al*. [8] compared restorative materials produced using various manufacturing technologies, including conventional, CAD/CAM, and 3D-printed resins. Their results demonstrated a positive correlation between increased filler-particle content and both Vickers microhardness and elastic modulus. Among the 3D-printed resins analyzed, the only resin indicated for permanent restorations and containing approximately 50% ceramic filler exhibited the highest values for these properties. Building on these findings, Şahin *et al*. [9] compared 3D-printed and CAD/CAM resins for permanent restorations, with manufacturer-reported inorganic filler contents of 30-50% and 75-86%, respectively. Although CAD/CAM materials exhibited higher elastic modulus and surface microhardness values, one 3D-printed resin achieved flexural strength statistically comparable to that of certain CAD/CAM materials. Similarly, a recent study reported that 3D-printed resins intended for permanent restorations exhibited fracture resistance comparable to hybrid CAD/CAM materials [10]. However, current evidence indicates that milled CAD/CAM resins generally provide superior mechanical performance compared to 3D-printed resins [11].

Post-curing is a critical stage in the manufacturing process of 3D-printed resins, as it determines the material’s final properties. During post-curing, residual monomers continue to convert, which directly affects the mechanical and surface characteristics of these resins [12]. Kim *et al*. [13] suggested that modifying post-curing protocols can optimize the performance of 3D-printed resins. The extent of this effect depends on factors such as formulation, inorganic filler content, post-curing unit, and protocol, all of which are important considerations for clinicians when selecting materials.

Therefore, this study aimed to compare the physicomechanical response of three 3D-printed resins intended for permanent restorations, each with distinct formulations and inorganic filler contents, when subjected to three material-specific post-curing durations: Half the manufacturer-recommended time, the recommended time, and twice the recommended time.

## Materials and Methods

*Study design and ethical considerations*

An *in vitro* experimental study was conducted. The study protocol was approved by a local Institutional Research Ethics Committee (SIDISI: 216301).

*Sample size*

Sample size calculations were performed independently for each test, including surface microhardness, flexural strength, and fracture resistance, using G*Power software version 3.1.3 (Heinrich Heine University, Düsseldorf, Germany). For all tests, analysis of variance was applied to assess main effects and interactions, with an effect size of 0.40, a significance level of 0.05, and a statistical power of 0.80, following Cohen’s recommendations as reported by Lakens [14].

The calculations indicated a minimum sample size of 79 specimens per test. To enhance statistical robustness, the sample size was increased to 90, divided into nine subgroups of 10 specimens each, based on three resin brands and three post-curing times. An additional reference group comprising a CAD/CAM nanohybrid resin (10 specimens) brought the total to 100 specimens per test.

*Materials and group allocation*

The specimens were allocated to four groups based on the material evaluated: Three 3D-printed resins intended for permanent restorations and one CAD/CAM resin, serving as the reference group (Figure 1). The materials evaluated were VarseoSmile TriniQ (BEGO, Bremen, Germany), Prizma 3D Bio Crown Diamond and Prizma 3D Bio Crown (Makertech Labs, São Paulo, Brazil), and Evolux Hybrid (Bluedent, Pirassununga, São Paulo, Brazil). Their main characteristics are described in Table 1.

Each 3D-printed resin was split into three subgroups by post-curing time (T1, T2, and T3), with 10 specimens per subgroup. The control group also had 10 specimens and was not subdivided. This grouping was consistent for all properties evaluated: Surface microhardness, flexural strength, and fracture resistance.

*Study specimens*

For the flexural strength test, bar-shaped specimens measuring 25 × 2 × 2 mm were fabricated in accordance with ISO 4049:2019. For the surface microhardness and fracture resistance tests, disc-shaped specimens measuring 10 mm in diameter and 2 mm in thickness were fabricated following the methodology described in previous studies [15] (Figure 2A).

*Digital specimen design*

The bar- and disc-shaped specimens were designed using the computer-aided design software Tinkercad (Autodesk Inc., San Rafael, CA, USA). The designs were then saved as Standard Tessellation Language (STL) files and imported into the slicing software for each 3D-printing system.

*Specimen fabrication*

Specimens were fabricated using 3D printing and post-curing systems recommended for or compatible with each resin, because compatibility among the material, printer, and post-curing protocol may affect the final properties of 3D-printed resins [16]. Within each material, the printer and post-curing unit were kept constant across the three post-curing times, allowing the response to post-curing duration to be evaluated. The manufacturing equipment and key technical parameters are detailed in Table 1.

VarseoSmile TriniQ was printed using an AccuFab-L4D printer (SHINING 3D), whereas Prizma 3D Bio Crown Diamond and Prizma 3D Bio Crown were printed using a Phrozen Sonic Mini 8K printer (Phrozen Tech Co., Ltd.). A CAD/CAM nanohybrid resin was used as the reference control material, and its specimens were produced using an M1 Abutment Mill (Zirkonzahn GmbH).

After printing, support structures were removed. Next, specimens were cleaned with 99% isopropyl alcohol using a CD-4820 digital ultrasonic cleaner (GT SONIC Co., Guangdong, China). Cleaning consisted of two consecutive 3-minute cycles in separate containers. Subsequently, the specimens were rinsed with fresh isopropyl alcohol and dried before post-curing.

*Post-curing times*

The post-curing units and their respective reference times varied according to the resin evaluated, as shown in Table 1. Experimental post-curing times were defined as follows: T1 was half the reference time, T2 was the reference time, and T3 was twice the reference time.

For VarseoSmile TriniQ, which had a reference time of two 5-minute cycles totaling 10 minutes, T1 was set to 5 minutes, T2 to 10 minutes, and T3 to 20 minutes.

For Prizma 3D Bio Crown Diamond, with a reference time of 20 minutes, T1 was 10 minutes, T2 was 20 minutes, and T3 was 40 minutes.

For Prizma 3D Bio Crown, which had a reference time of 30 minutes, T1 was 15 minutes, T2 was 30 minutes, and T3 was 60 minutes.

Following post-curing, all specimens were inspected with an SM-4TZ-144A trinocular stereomicroscope (AmScope, Irvine, CA, USA) to identify printing-related surface defects, including irregularities, bubbles, cracks, and areas of incomplete polymerization. Specimens presenting any of these alterations were excluded and replaced. Subsequently, the disc-shaped specimens were stored in opaque containers to prevent light exposure. After 24 h, they were manually polished using progressively finer abrasive papers. Following polishing, their dimensions were verified using a digital caliper with a measurement range of 0-150 mm (INGCO Tools Co., Ltd., Suzhou, China), and the surfaces were re-examined under the same stereomicroscope to confirm the absence of defects. Specimens presenting dimensional deviations or polishing-related surface defects were excluded and replaced. Finally, the accepted specimens were prepared for testing.

*Surface microhardness test*

Surface microhardness was measured on the top and bottom surfaces of each disc-shaped specimen using a microhardness tester equipped with a Vickers indenter (AMH55; LECO, St. Joseph, MI, USA). Three indentations were made on each surface, for a total of six indentations per specimen, under a 50-g load applied for 30 s [17]. The resulting values were averaged to obtain a single microhardness value for each specimen (Figure 2B).

*Flexural strength test*

Flexural strength was evaluated using a three-point bending test in a universal testing machine (OM 150; Odeme Dental Research, Joaçaba, SC, Brazil). The specimens were positioned on two supports with a 20 mm span. A vertical compressive load was applied at the center of each specimen at a crosshead speed of 1 mm/min until fracture occurred. The maximum load at fracture was used to calculate the flexural strength (σ) in MPa [17] (Figure 2C).

*Fracture resistance test*

Fracture resistance was evaluated using a universal testing machine (OM 150; Odeme Dental Research, Joaçaba, SC, Brazil). A vertical compressive load was applied at the center of each specimen at a crosshead speed of 1 mm/min until fracture occurred. The maximum load recorded at fracture was considered the fracture resistance and was expressed in newtons (N) (Figure 2D).

*Statistical analysis*

Statistical analyses were conducted using Stata version 17 (StataCorp LLC, College Station, TX, USA). Descriptive statistics are presented as mean ± standard deviation. The effects of material, post-curing time, and their interaction on surface microhardness, flexural strength, and fracture resistance were assessed using two-way analysis of variance (two-way ANOVA). When significant differences were observed, Bonferroni post hoc tests were applied for multiple comparisons. The significance threshold was set at *α*=0.05. The CAD/CAM material was used solely as a reference group and was excluded from the factorial analysis. Graphs were created using GraphPad Prism version 10.0 (GraphPad Software, Boston, MA, USA).

## Results

Microhardness, flexural strength, and fracture resistance varied depending on both the material and the post-curing time evaluated (Table 2). For the 3D-printed resins, microhardness increased with longer post-curing times, with the highest values observed at T3 for Bio Crown Diamond (23.59 ± 1.58 VHN) and VarseoSmile TriniQ (23.47 ± 1.17 VHN). VarseoSmile TriniQ demonstrated the highest flexural strength at all post-curing times, while Bio Crown exhibited the lowest values. Fracture resistance also increased with post-curing time for all three 3D-printed resins, with VarseoSmile TriniQ-T3 achieving the highest value (933.14 ± 20.77 N), followed by Bio Crown Diamond-T3 (880.79 ± 17.16 N). The CAD/CAM reference material, Evolux Hybrid, showed a surface microhardness of 28.12 ± 2.79 VHN and a flexural strength of 112.86 ± 1.42 MPa. In the fracture resistance test, all Evolux Hybrid specimens reached the testing machine's maximum recording capacity (980 N) without fracturing. As a result, their actual fracture resistance values could not be determined and were reported as greater than 980 N.

Table 3 presents the results of the two-way ANOVA. Both material and post-curing time significantly influenced all three properties (*p*<0.001), although their relative contributions varied. Material accounted for the majority of the variability in Vickers microhardness (partial η²=0.914), flexural strength (partial η²=0.882), and fracture resistance (partial η²=0.949). The effect of post-curing time was most pronounced for fracture resistance (partial η²=0.990). The interaction between material and time was relevant for flexural strength (partial η²=0.342) and fracture resistance (partial η²=0.799), but not for Vickers microhardness (*p*=0.191).

Post-curing time had a significant impact on the physicomechanical properties of the three resins, with the extent of these effects varying by material and property (Figure 3). For all materials, T1, representing half the recommended post-curing time, generally resulted in the lowest property values. In Bio Crown, the manufacturer-recommended post-curing time (T2) resulted in significant increases in flexural strength and fracture resistance compared with T1 (*p*<0.05). Vickers microhardness in Bio Crown differed significantly only between T1 and T3. No significant differences were detected between T2 and T3 for any evaluated property (*p*>0.05). In VarseoSmile TriniQ, fracture resistance increased significantly across all post-curing times (*p*<0.05). Vickers microhardness was significantly higher at T3 than at T1 and T2, while flexural strength differed significantly only between T1 and T3 (*p*<0.05). Bio Crown Diamond exhibited the most consistent progressive increases, with flexural strength and fracture resistance rising significantly from T1 to T2 and from T2 to T3 (*p*<0.05). Vickers microhardness increased significantly from T1 to T2, with no further significant increase between T2 and T3. Detailed pairwise comparisons, including mean differences, 95% confidence intervals, and adjusted *p* values, are presented in Supplementary Tables S1A-S1C (https://www.medicinaoral.com/medoralfree01/64561/Suppl1.pdf)

## Discussion

This study evaluated the effects of three post-curing times on the surface microhardness, flexural strength, and fracture resistance of three 3D-printed resins intended for permanent restorations, each with a different inorganic filler content. A CAD/CAM nanohybrid resin was used as a reference material. The findings indicate that both material type and post-curing duration significantly influenced the properties evaluated. Although the values generally increased with longer post-curing times, the magnitude of this improvement varied according to the material and property evaluated.

Surface microhardness and flexural strength increased with longer post-curing times. The highest values were observed at T3, which corresponded to twice the manufacturer-recommended time. This trend is consistent with the findings of Kim *et al*. [13], who reported similar increases in both properties in 3D-printed resins for crowns and bridges after prolonged post-curing. Karademir *et al*. [12] also observed higher Vickers microhardness in 3D-printed resins for permanent restorations after longer post-curing times. Similarly, Alharbi *et al*. [18] reported significant increases in surface microhardness and flexural strength with prolonged post-curing, along with a positive correlation between these properties.

A comparable trend was observed for fracture resistance, with the highest values measured at T3. This result aligns with the findings of Korkmaz *et al*. [19], who reported that extended post-curing enhanced fracture resistance in 3D-printed resins for permanent restorations. In contrast, Hatem *et al*. [20] did not observe significant differences in the fracture resistance of crowns produced from a ceramic-filled 3D-printed hybrid resin with increased post-curing time. This inconsistency may be attributed to methodological differences, as the studies employed different materials and post-curing intervals. Specifically, Hatem *et al*. assessed only two post-curing durations using anatomically shaped crowns subjected to thermal aging, whereas the present study examined three material-specific post-curing times and utilized disc-shaped specimens. This approach enabled a direct comparison of the three resins under standardized dimensional and loading conditions, thereby minimizing variability related to restoration anatomy.

However, this configuration does not reproduce the stress distribution of an anatomically shaped restoration or the clinical conditions to which it would be subjected. Although these methodological differences limit direct comparisons with previous studies, the results provide a valuable reference for the behavior of these materials in relation to the variables evaluated.

These results show that fracture resistance was also sensitive to variations in post-curing time. However, because the present study did not evaluate the degree of conversion or the polymer network characteristics, the mechanisms underlying this behavior cannot be established. Regarding the materials, VarseoSmile TriniQ exhibited the highest flexural strength and fracture resistance among the materials evaluated. Together with Bio Crown Diamond, it showed comparable surface microhardness at T3. This finding is consistent with that of Tzimas *et al*. [21], who characterized VarseoSmile TriniQ as a ceramic-filled hybrid resin suitable for permanent restorations and reported favorable surface properties, including microhardness comparable to that of hybrid CAD/CAM materials. Gültekin *et al*. [22] also reported flexural strength values ranging from 103.75 to 119.25 MPa and a microhardness of approximately 34 VHN, determined using the manufacturer’s protocol. The T2 values obtained in the present study were consistent with previous reports, whereas the T3 values were higher, indicating that prolonged post-curing may further improve mechanical performance.

Hartavi *et al*. [23] reported that VarseoSmile TriniQ contains a methacrylate-based matrix and approximately 50 wt% inorganic filler. In the present study, VarseoSmile TriniQ exhibited the highest flexural strength and fracture resistance values among the 3D-printed resins evaluated. Kim *et al*. [24] demonstrated that the physicomechanical properties of 3D-printed hybrid resins also depend on microstructural factors, including filler particle distribution, matrix-filler interfacial quality, and the presence of internal defects. Therefore, although the filler content of TriniQ may play a role, other material-specific characteristics also influence its performance. Abdulai *et al*. [25] found that post-printing processing may modify the optical, surface, and mechanical properties of this material. Although their study focused on post-printing cleaning rather than on post-curing time, their findings suggest that processing conditions and the material's compositional and microstructural characteristics jointly determine its final behavior.

The available evidence regarding Bio Crown Diamond remains limited. For example, the study by Dos Santos Brito *et al*. [26] is among the few investigations to include this material and described it as a 3D-printable nanohybrid resin for permanent restorations. According to the manufacturer, more than 52 wt% of its ceramic fillers are silanized. At T3, Bio Crown Diamond achieved Vickers microhardness values comparable to those of VarseoSmile TriniQ. However, these microhardness values were not accompanied by similarly high flexural strength or fracture resistance values. This finding is consistent with that of Kim *et al*. [24], who concluded that the performance of 3D-printed hybrid resins depends not only on their inorganic filler content but also on the characteristics of the resin matrix, filler distribution, and the quality of the matrix-filler interface.

In contrast, Bio Crown showed the lowest values for all three properties. This material has an organic matrix composed primarily of UDMA and a lower inorganic content than the other 3D-printed resins. According to the manufacturer’s information reported by Soares *et al*. [27], Prizma 3D Bio Crown contains more than 10% methacrylate monomers, less than 0.5% titanium dioxide, less than 2% silanized zirconia, up to 8% amorphous silica, and less than 15% ceramic fillers. Therefore, its inorganic content may reach approximately 25.5 wt%. In the present study, Bio Crown exhibited the lowest values for all three properties evaluated. At T3, surface microhardness and fracture resistance were higher than at T1, whereas flexural strength showed little improvement compared with T2. This finding is consistent with that of Dzhondrova *et al*. [28], who found that mechanical properties respond differently to post-curing, with an increase in microhardness but little improvement in flexural strength after prolonged polymerization. Similarly, Souza *et al*. [29] identified irregular and porous surfaces, as well as fractures originating from defects located in areas of stress concentration. According to the authors, these voids may result from inadequate mixing of the resin components and limitations in polymerization, which may weaken the material's mechanical performance. However, because no microstructural characterization was performed, the potential influence of filler distribution, matrix-filler interactions, porosity, and internal defects on the behavior of the materials should be interpreted with caution, as it was not confirmed in the present study.

In the present study, Evolux Hybrid was selected as the CAD/CAM reference material because, according to its manufacturer, it contains 45-50 wt% silanized glass nanoparticles. This inorganic filler content is similar to that of the 3D-printed resins with the highest filler percentages among the materials evaluated. Evolux Hybrid exhibited the highest Vickers microhardness among the materials evaluated, whereas its flexural strength was within the range observed for VarseoSmile TriniQ. In the fracture resistance test, all specimens reached the testing machine's maximum recording limit of 980 N without fracturing. Therefore, their actual fracture resistance values could not be determined and were above 980 N. Consequently, this result indicates that the specimens withstood higher loads than those of the other groups; however, the magnitude of the difference relative to the 3D-printed resins could not be quantified.

A previous study by our research group showed that 1.5-mm-thick occlusal veneers fabricated from Evolux Hybrid achieved fracture resistance values of up to 915 N [30]. Unlike the present study, that investigation used anatomically shaped specimens cemented onto three types of dental substrates, which may have influenced the values obtained for this property. Despite the differences in experimental design, both studies indicate favorable mechanical behavior for this material.

From a clinical perspective, the physicomechanical improvements observed after longer post-curing provide comparative *in vitro* data on the material-specific response. Although these findings may suggest mechanical advantages, they do not demonstrate that doubling the post-curing time improves clinical longevity, as performance will also depend on restoration geometry and thickness, stress distribution, dental substrate, cementation, and the functional dynamics of the oral environment. Therefore, the *in vitro* results are promising but do not guarantee greater clinical longevity without additional evidence. 

In addition to the limitations inherent to the *in vitro* design, the present study did not evaluate the degree of conversion, characterize the internal microstructure, or analyze the distribution of inorganic fillers. Consequently, mechanisms related to these variables should be interpreted as possible explanations rather than processes demonstrated in the present study. Furthermore, no artificial aging protocols, such as thermocycling, prolonged water storage, or cyclic fatigue, were applied; therefore, the long-term stability of the evaluated properties under simulated oral conditions could not be assessed.

Furthermore, the printing and post-curing systems were selected according to the manufacturer’s recommendations or their established compatibility with each resin. They were kept constant across the three post-curing times evaluated. Therefore, variability associated with these systems was controlled for when analyzing the effect of post-curing time within each material. However, because the same system was not used for all resins, comparisons between materials reflect each resin's performance within its corresponding manufacturing workflow and do not permit the observed differences to be attributed exclusively to material composition. Moreover, all Evolux Hybrid specimens exceeded the maximum recording capacity of the universal testing machine without fracturing, thereby preventing determination of their actual maximum values and limiting their quantitative comparison with the 3D-printed resins. Future studies should incorporate complementary chemical and microstructural analyses, as well as aging and cyclic loading protocols and experimental designs that more closely reproduce clinical conditions.

## Conclusions

The present study found that post-curing time influenced the physicomechanical properties of 3D-printed resins intended for permanent restorations, with the response depending on the material and property evaluated. VarseoSmile TriniQ exhibited the highest flexural strength and fracture resistance. In contrast, Bio Crown Diamond achieved surface microhardness comparable to that of VarseoSmile TriniQ only after post-curing for twice the recommended duration.

## Supplementary Material

Supplementary 1

## Figures and Tables

**Table 1:** Table 1: Characteristics of the materials used in the study.

**Material**	**Composition**	**Printing and post-curing parameters**
VarseoSmile TriniQ (BEGO, Bremen, Germany)	Methacrylate-based matrix containing additives and photoinitiators, reinforced with ~50 wt% inorganic filler composed of ceramic fillers and silanized nanohybrid glass particles.	Compatible with LCD/DLP printing and photopolymerization at 385-405 nm. Printer: AccuFab-L4D (SHINING 3D, Hangzhou, China). Layer thickness: 0.05 mm (50 µm). Post-curing unit: FabCure 2 (SHINING 3D, Hangzhou, China), equipped with 385-405 nm UV LEDs, a power output of 70 W, and temperature control up to 60 °C. Recommended protocol: Two 5-min cycles at 20 °C.
PriZma 3D Bio Crown Diamond (Makertech Labs, São Paulo, Brazil)	Photopolymerizable nanohybrid resin composed of oligomers, monomers, photoinitiators, UV absorbers, fillers, and pigments, containing >52% silanized ceramic fillers.	Compatible with LCD/DLP printing and photopolymerization at 385-405 nm. Printer: Phrozen Sonic Mini 8K (Phrozen Tech Co., Ltd., Hsinchu, Taiwan). Post-curing unit: Cure Luna (Phrozen Tech Co., Ltd., Hsinchu, Taiwan), equipped with 405 nm UV LEDs. Recommended post-curing time: 20 min.
PriZma 3D Bio Crown (Makertech Labs, São Paulo, Brazil)	Resin with an organic matrix composed of methacrylate monomers and UDMA, containing ≤25.5 wt% inorganic filler consisting of ceramic fillers, amorphous silica, silanized zirconia, and titanium dioxide.	Compatible with LCD/DLP printing and photopolymerization at 385-405 nm. Printer: Phrozen Sonic Mini 8K (Phrozen Tech Co., Ltd., Hsinchu, Taiwan). Post-curing unit: Cure Luna (Phrozen Tech Co., Ltd., Hsinchu, Taiwan), equipped with 405 nm UV LEDs. Recommended post-curing time: 30 min.
Evolux Hybrid (Bluedent, Pirassununga, São Paulo, Brazil)	CAD/CAM nanohybrid resin with a polymeric resin matrix and 45-50% silanized inorganic glass nanoparticles.	Prefabricated CAD/CAM material. The specimens were obtained by milling using an M1 Abutment Mill (Zirkonzahn GmbH, Gais, Italy). 3D printing and UV post-curing: Not applicable.

**Table 2:** Table 2: Mean values and standard deviations of the physicomechanical properties evaluated according to material and post-curing time.

**Material**	**Post-curing time**	**Vickers microhardness (VHN)**	**Flexural strength (MPa)**	**Fracture resistance (N)**
Bio Crown	T1	10.82 ± 1.25	47.98 ± 3.50	322.36 ± 21.84
	T2	12.76 ± 0.95	63.89 ± 3.95	622.74 ± 28.40
	T3	13.36 ± 1.36	58.41 ± 4.02	638.16 ± 9.53
Bio Crown Diamond	T1	19.41 ± 1.40	49.88 ± 8.34	400.55 ± 7.47
	T2	21.89 ± 2.07	68.33 ± 11.10	835.10 ± 36.86
	T3	23.59 ± 1.58	95.88 ± 22.22	880.79 ± 17.16
VarseoSmile TriniQ	T1	20.34 ± 1.28	110.87 ± 6.02	388.45 ± 12.19
	T2	21.31 ± 1.51	118.29 ± 7.01	852.23 ± 23.65
	T3	23.47 ± 1.17	128.14 ± 11.35	933.14 ± 20.77
Evolux Hybrid CAD/CAM	-	28.12 ± 2.79	112.86 ± 1.42	>980

T1: Half the manufacturer-recommended post-curing time; T2: The manufacturer-recommended post-curing time; T3: Twice the manufacturer-recommended post-curing time. Values are expressed as mean ± standard deviation. Evolux Hybrid CAD/CAM was included as a reference control; all specimens reached the maximum recording capacity of the universal testing machine (980 N) without fracturing

**Table 3:** Table 3: Two-way ANOVA results for the evaluated mechanical properties.

**Properties**	**Effect**	*df*	*F*	*p* ** value**	**η²** *p*
Vickers microhardness	Material	2, 81	429.40	<0.001	0.914
	Time	2, 81	39.79	<0.001	0.496
	Material × Time	4, 81	1.57	0.191	0.072
Flexural strength	Material	2, 81	303.80	<0.001	0.882
	Time	2, 81	43.38	<0.001	0.517
	Material × Time	4, 81	10.52	<0.001	0.342
Fracture resistance	Material	2, 81	753.50	<0.001	0.949
	Time	2, 81	3854.00	<0.001	0.990
	Material × Time	4, 81	80.27	<0.001	0.799

df: Degree of freedom; η^2^p: Partial eta squared.

**Figure 1:**
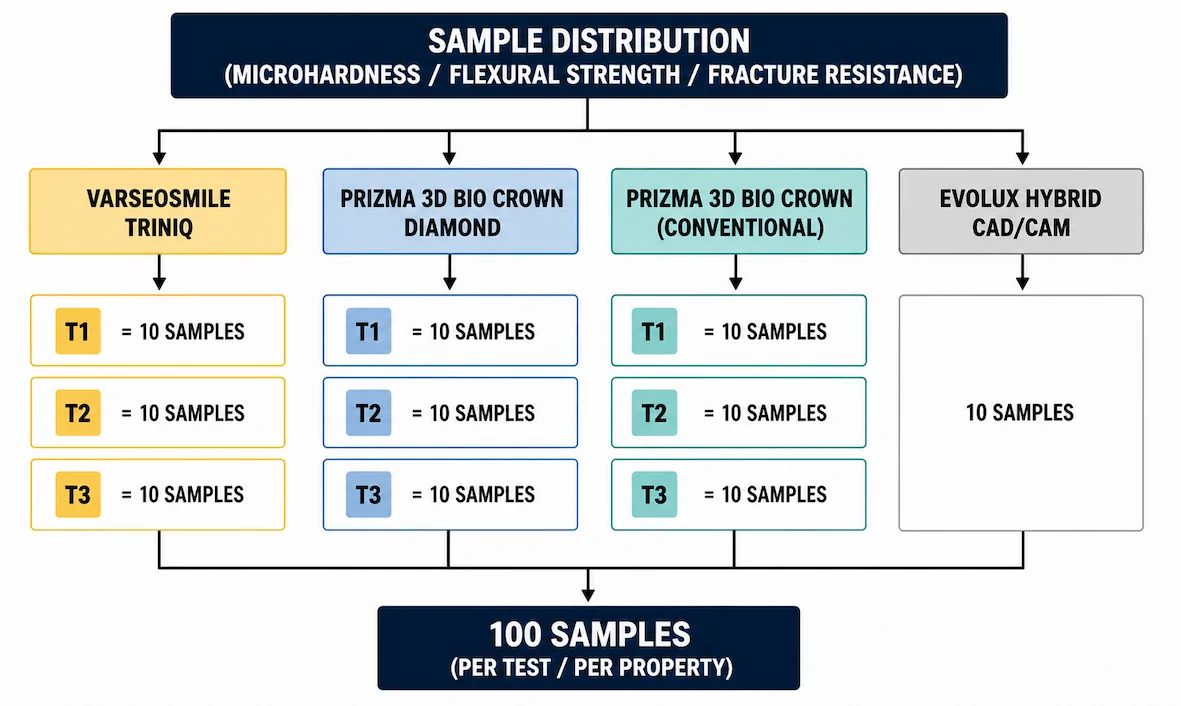
Fig. 1: Experimental group and specimen distribution.

**Figure 2:**
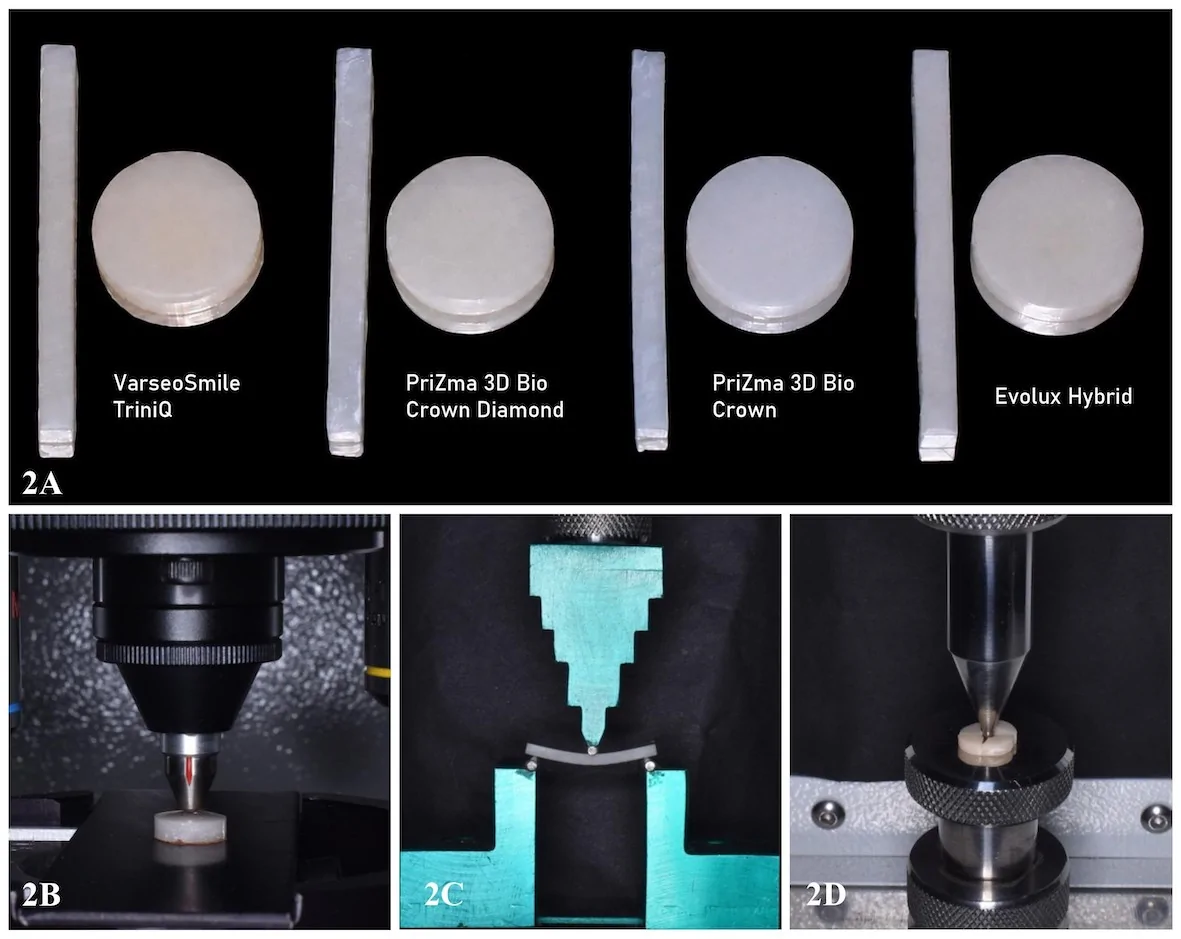
Fig. 2: Specimens and physicomechanical evaluation procedures. A: Bar- and disc-shaped specimens used in the study; B: Vickers microhardness assessment; C: Flexural strength test; and D: Fracture resistance test.

**Figure 3:**
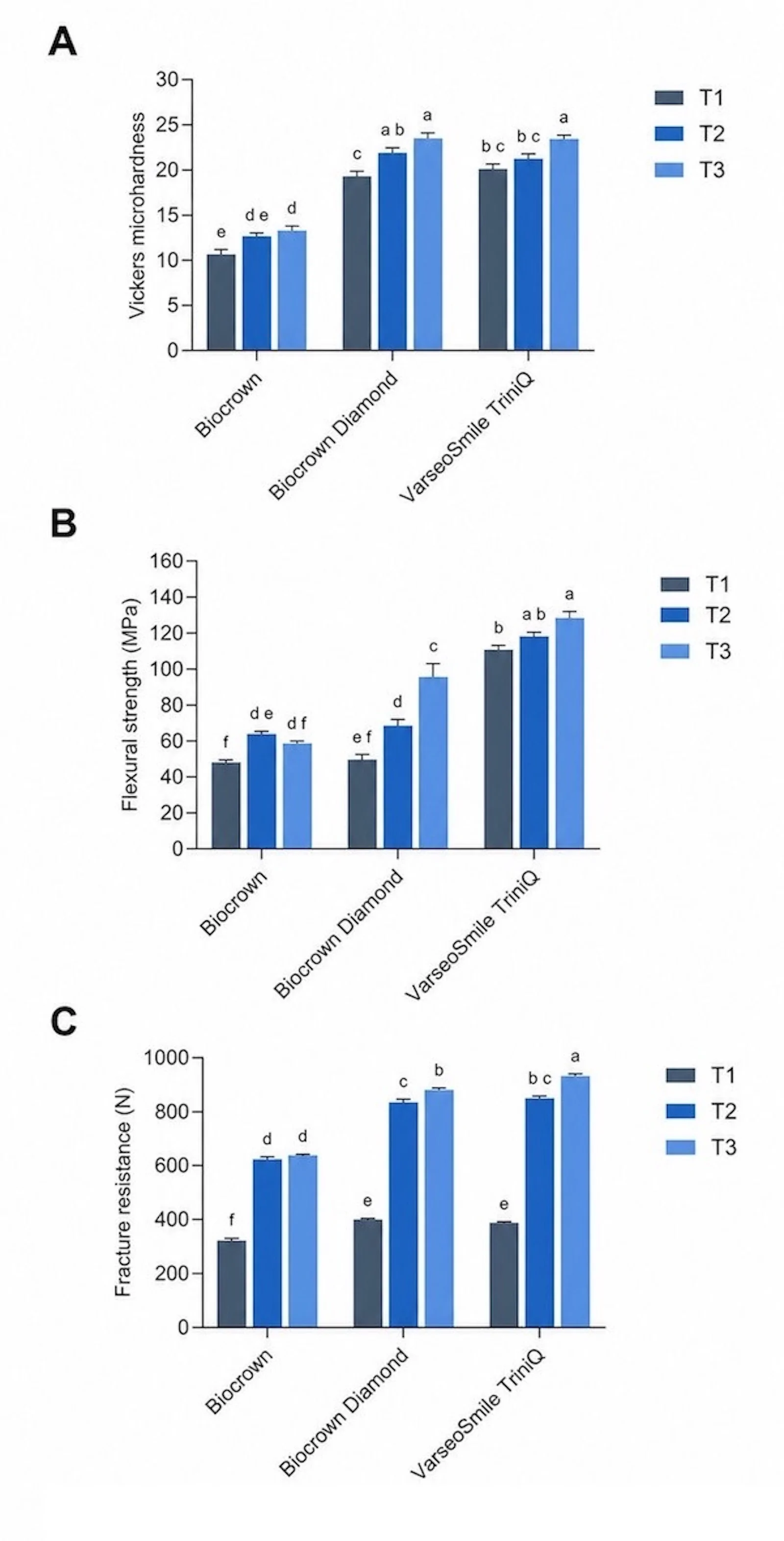
Fig. 3: Effect of post-curing time on Vickers microhardness (A), flexural strength (B), and fracture resistance (C) of three 3D-printed resins. Different letters indicate statistically significant differences among post-curing times within each material (p<0.05).

## Data Availability

Declared none.
