# Supplementary material for: Effect of different post-curing times on the physicomechanical properties of 3D-printed resins intended for permanent restorations
Source: J Clin Exp Dent. 2026 Sep 15;18(10):e1164–72. doi: 10.4317/jced.64561 (PMC13739079; doi:10.4317/jced.64561)
Supplement: Supplementary file 1 [file jced-18-10-e1164-s001.pdf]

Supplementary Table S1A. Bonferroni-adjusted pairwise comparisons of Vickers microhardness among material × time combinations

Comparison	Mean difference	95% CI	Adjusted p value
Biocrown Diamond T1 vs T2	-2.48	-4.59 to -0.37	**0.0075**
Biocrown Diamond T1 vs T3	-4.18	-6.29 to -2.07	**<0.0001**
Biocrown Diamond T1 vs TriniQ T1	-0.93	-3.04 to 1.18	>0.9999
Biocrown Diamond T1 vs TriniQ T2	-1.90	-4.01 to 0.21	0.1386
Biocrown Diamond T1 vs TriniQ T3	-4.06	-6.17 to -1.95	**<0.0001**
Biocrown Diamond T1 vs Biocrown T1	8.59	6.48 to 10.70	**<0.0001**
Biocrown Diamond T1 vs Biocrown T2	6.65	4.54 to 8.76	**<0.0001**
Biocrown Diamond T1 vs Biocrown T3	6.05	3.94 to 8.16	**<0.0001**
Biocrown Diamond T2 vs T3	-1.70	-3.81 to 0.41	0.3366
Biocrown Diamond T2 vs TriniQ T1	1.55	-0.56 to 3.66	0.6268
Biocrown Diamond T2 vs TriniQ T2	0.58	-1.53 to 2.69	>0.9999
Biocrown Diamond T2 vs TriniQ T3	-1.58	-3.69 to 0.53	0.5552
Biocrown Diamond T2 vs Biocrown T1	11.07	8.96 to 13.18	**<0.0001**
Biocrown Diamond T2 vs Biocrown T2	9.13	7.02 to 11.24	**<0.0001**
Biocrown Diamond T2 vs Biocrown T3	8.53	6.42 to 10.64	**<0.0001**
Biocrown Diamond T3 vs TriniQ T1	3.25	1.14 to 5.36	**<0.0001**
Biocrown Diamond T3 vs TriniQ T2	2.28	0.17 to 4.39	**0.0217**
Biocrown Diamond T3 vs TriniQ T3	0.12	-1.99 to 2.23	>0.9999
Biocrown Diamond T3 vs Biocrown T1	12.77	10.66 to 14.88	**<0.0001**
Biocrown Diamond T3 vs Biocrown T2	10.83	8.72 to 12.94	**<0.0001**
Biocrown Diamond T3 vs Biocrown T3	10.23	8.12 to 12.34	**<0.0001**
TriniQ T1 vs T2	-0.97	-3.08 to 1.14	>0.9999
TriniQ T1 vs T3	-3.13	-5.24 to -1.02	**0.0002**
TriniQ T1 vs Biocrown T1	9.52	7.41 to 11.63	**<0.0001**
TriniQ T1 vs Biocrown T2	7.58	5.47 to 9.69	**<0.0001**
TriniQ T1 vs Biocrown T3	6.98	4.87 to 9.09	**<0.0001**
TriniQ T2 vs T3	-2.16	-4.27 to -0.05	**0.0398**

Comparison	Mean difference	95% CI	Adjusted p value
TriniQ T2 vs Biocrown T1	10.49	8.38 to 12.60	**<0.0001**
TriniQ T2 vs Biocrown T2	8.55	6.44 to 10.66	**<0.0001**
TriniQ T2 vs Biocrown T3	7.95	5.84 to 10.06	**<0.0001**
TriniQ T3 vs Biocrown T1	12.65	10.54 to 14.76	**<0.0001**
TriniQ T3 vs Biocrown T2	10.71	8.60 to 12.82	**<0.0001**
TriniQ T3 vs Biocrown T3	10.11	8.00 to 12.22	**<0.0001**
Biocrown T1 vs T2	-1.94	-4.05 to 0.17	0.1152
Biocrown T1 vs T3	-2.54	-4.65 to -0.43	**0.0054**
Biocrown T2 vs T3	-0.60	-2.71 to 1.51	>0.9999

Note. Mean differences were calculated as the first group minus the second group. Confidence intervals and p values were adjusted using Bonferroni's multiple-comparison procedure. Bold p values indicate statistically significant differences ($p < 0.05$). T1, T2, and T3 represent the respective evaluation times.

Supplementary Table S1B. Bonferroni-adjusted pairwise comparisons of flexural strength among material × time combinations.

Comparison	Mean difference (MPa)	95% CI	Adjusted p value
Biocrown Diamond T1 vs T2	−18.45	−33.05 to −3.85	**0.0038**
Biocrown Diamond T1 vs T3	−46.00	−60.60 to −31.40	**<0.0001**
Biocrown Diamond T1 vs TriniQ T1	−60.99	−75.59 to −46.39	**<0.0001**
Biocrown Diamond T1 vs TriniQ T2	−68.41	−83.01 to −53.81	**<0.0001**
Biocrown Diamond T1 vs TriniQ T3	−78.26	−92.86 to −63.66	**<0.0001**
Biocrown Diamond T1 vs Biocrown T1	1.90	−12.70 to 16.50	>0.9999
Biocrown Diamond T1 vs Biocrown T2	−14.01	−28.61 to 0.59	0.0703
Biocrown Diamond T1 vs Biocrown T3	−8.53	−23.13 to 6.07	0.6414
Biocrown Diamond T2 vs T3	−27.55	−42.15 to −12.95	**<0.0001**
Biocrown Diamond T2 vs TriniQ T1	−42.54	−57.14 to −27.94	**<0.0001**

Comparison	Mean difference (MPa)	95% CI	Adjusted p value
Biocrown Diamond T2 vs TriniQ T2	−49.96	−64.56 to −35.36	**<0.0001**
Biocrown Diamond T2 vs TriniQ T3	−59.81	−74.41 to −45.21	**<0.0001**
Biocrown Diamond T2 vs Biocrown T1	20.35	5.75 to 34.95	**0.0009**
Biocrown Diamond T2 vs Biocrown T2	4.44	−10.16 to 19.04	0.9876
Biocrown Diamond T2 vs Biocrown T3	9.92	−4.68 to 24.52	0.4380
Biocrown Diamond T3 vs TriniQ T1	−14.99	−29.59 to −0.39	**0.0397**
Biocrown Diamond T3 vs TriniQ T2	−22.41	−37.01 to −7.81	**0.0002**
Biocrown Diamond T3 vs TriniQ T3	−32.26	−46.86 to −17.66	**<0.0001**
Biocrown Diamond T3 vs Biocrown T1	47.90	33.30 to 62.50	**<0.0001**
Biocrown Diamond T3 vs Biocrown T2	31.99	17.39 to 46.59	**<0.0001**
Biocrown Diamond T3 vs Biocrown T3	37.47	22.87 to 52.07	**<0.0001**
TriniQ T1 vs T2	−7.42	−22.02 to 7.18	0.7914
TriniQ T1 vs T3	−17.27	−31.87 to −2.67	**0.0090**
TriniQ T1 vs Biocrown T1	62.89	48.29 to 77.49	**<0.0001**
TriniQ T1 vs Biocrown T2	46.98	32.38 to 61.58	**<0.0001**
TriniQ T1 vs Biocrown T3	52.46	37.86 to 67.06	**<0.0001**
TriniQ T2 vs T3	−9.85	−24.45 to 4.75	0.4479
TriniQ T2 vs Biocrown T1	70.31	55.71 to 84.91	**<0.0001**
TriniQ T2 vs Biocrown T2	54.40	39.80 to 69.00	**<0.0001**
TriniQ T2 vs Biocrown T3	59.88	45.28 to 74.48	**<0.0001**
TriniQ T3 vs Biocrown T1	80.16	65.56 to 94.76	**<0.0001**
TriniQ T3 vs Biocrown T2	64.25	49.65 to 78.85	**<0.0001**
TriniQ T3 vs Biocrown T3	69.73	55.13 to 84.33	**<0.0001**
Biocrown T1 vs T2	−15.91	−30.51 to −1.31	**0.0223**
Biocrown T1 vs T3	−10.43	−25.03 to 4.17	0.3685
Biocrown T2 vs T3	5.48	−9.12 to 20.08	0.9551

Note. Mean differences were calculated as the first group minus the second group. Confidence intervals and p values were adjusted using Bonferroni's multiple-comparison procedure. Bold p values indicate statistically significant differences ($p < 0.05$). T1, T2, and T3 represent the respective evaluation times.

Supplementary Table S1C. Bonferroni-adjusted pairwise comparisons of fracture resistance among material × time combinations

Comparison	Mean difference (N)	95% CI	Adjusted p value
Biocrown T1 vs T2	−300.40	−331.30 to −269.50	**<0.0001**
Biocrown T1 vs T3	−315.80	−346.70 to −284.90	**<0.0001**
Biocrown T1 vs Biocrown Diamond T1	−78.19	−109.10 to −47.32	**<0.0001**
Biocrown T1 vs Biocrown Diamond T2	−512.70	−543.60 to −481.90	**<0.0001**
Biocrown T1 vs Biocrown Diamond T3	−558.40	−589.30 to −527.60	**<0.0001**
Biocrown T1 vs TriniQ T1	−66.09	−96.96 to −35.22	**<0.0001**
Biocrown T1 vs TriniQ T2	−529.90	−560.70 to −499.00	**<0.0001**
Biocrown T1 vs TriniQ T3	−610.80	−641.70 to −579.90	**<0.0001**
Biocrown T2 vs T3	−15.42	−46.29 to 15.45	0.8064
Biocrown T2 vs Biocrown Diamond T1	222.20	191.30 to 253.10	**<0.0001**
Biocrown T2 vs Biocrown Diamond T2	−212.40	−243.20 to −181.50	**<0.0001**
Biocrown T2 vs Biocrown Diamond T3	−258.10	−288.90 to −227.20	**<0.0001**
Biocrown T2 vs TriniQ T1	234.30	203.40 to 265.20	**<0.0001**
Biocrown T2 vs TriniQ T2	−229.50	−260.40 to −198.60	**<0.0001**
Biocrown T2 vs TriniQ T3	−310.40	−341.30 to −279.50	**<0.0001**
Biocrown T3 vs Biocrown Diamond T1	237.60	206.70 to 268.50	**<0.0001**
Biocrown T3 vs Biocrown Diamond T2	−196.90	−227.80 to −166.10	**<0.0001**
Biocrown T3 vs Biocrown Diamond T3	−242.60	−273.50 to −211.80	**<0.0001**
Biocrown T3 vs TriniQ T1	249.70	218.80 to 280.60	**<0.0001**
Biocrown T3 vs TriniQ T2	−214.10	−244.90 to −183.20	**<0.0001**
Biocrown T3 vs TriniQ T3	−295.00	−325.90 to −264.10	**<0.0001**
Biocrown Diamond T1 vs T2	−434.60	−465.40 to −403.70	**<0.0001**

Comparison	Mean difference (N)	95% CI	Adjusted p value
Biocrown Diamond T1 vs T3	−480.20	−511.10 to −449.40	**<0.0001**
Biocrown Diamond T1 vs TriniQ T1	12.10	−18.77 to 42.97	0.9426
Biocrown Diamond T1 vs TriniQ T2	−451.70	−482.60 to −420.80	**<0.0001**
Biocrown Diamond T1 vs TriniQ T3	−532.60	−563.50 to −501.70	**<0.0001**
Biocrown Diamond T2 vs T3	−45.69	−76.56 to −14.82	**0.0003**
Biocrown Diamond T2 vs TriniQ T1	446.70	415.80 to 477.50	**<0.0001**
Biocrown Diamond T2 vs TriniQ T2	−17.13	−48.00 to 13.74	0.7023
Biocrown Diamond T2 vs TriniQ T3	−98.04	−128.90 to −67.17	**<0.0001**
Biocrown Diamond T3 vs TriniQ T1	492.30	461.50 to 523.20	**<0.0001**
Biocrown Diamond T3 vs TriniQ T2	28.56	−2.31 to 59.43	0.0925
Biocrown Diamond T3 vs TriniQ T3	−52.35	−83.22 to −21.48	**<0.0001**
VarseoSmile TriniQ T1 vs T2	−463.80	−494.70 to −432.90	**<0.0001**
VarseoSmile TriniQ T1 vs T3	−544.70	−575.60 to −513.80	**<0.0001**
VarseoSmile TriniQ T2 vs T3	−80.91	−111.80 to −50.04	**<0.0001**

Note. Mean differences were calculated as the first group minus the second group. Confidence intervals and p values were adjusted using Bonferroni's multiple-comparison procedure. Bold p values indicate statistically significant differences ($p < 0.05$). T1, T2, and T3 represent the respective evaluation times.
